# Supplementary material for: Unveiling the Bioactive Potential of the Invasive Jellyfish Phyllorhiza punctata Through Integrative Transcriptomic and Proteomic Analyses
Source: Biomolecules. 2025 Aug 4;15(8):1121. doi: 10.3390/biom15081121 (PMC12383608; doi:10.3390/biom15081121)
Supplement: Supplementary file 1 [file biomolecules-15-01121-s001.zip › Suplementary Material/FileS3/PEPFOLD4/XP_065055150_4289/clusters.html]

 PEP-FOLD clusters


Download archive of all models  here   

|  |  |  |
| --- | --- | --- |
| # | file | sOPEP |
| # cluster 1 - Size: 1 |  |  |
|  | XP\_065055150\_4289-model1 | -15.2927 |
| # cluster 2 - Size: 1 |  |  |
|  | XP\_065055150\_4289-model2 | -14.7009 |
| # cluster 3 - Size: 2 |  |  |
|  | XP\_065055150\_4289-model3 | -14.4688 |
|  | XP\_065055150\_4289-model3.1 | -9.86528 |
| # cluster 4 - Size: 1 |  |  |
|  | XP\_065055150\_4289-model4 | -9.54558 |
| # cluster 5 - Size: 1 |  |  |
|  | XP\_065055150\_4289-model5 | -9.17895 |
| # cluster 6 - Size: 1 |  |  |
|  | XP\_065055150\_4289-model6 | -8.72295 |
| # cluster 7 - Size: 2 |  |  |
|  | XP\_065055150\_4289-model7 | -8.59819 |
|  | XP\_065055150\_4289-model7.1 | -7.2797 |
| # cluster 8 - Size: 1 |  |  |
|  | XP\_065055150\_4289-model8 | -8.33991 |
| # cluster 9 - Size: 2 |  |  |
|  | XP\_065055150\_4289-model9 | -8.3011 |
|  | XP\_065055150\_4289-model9.1 | -7.79093 |
| # cluster 10 - Size: 2 |  |  |
|  | XP\_065055150\_4289-model10 | -8.2488 |
|  | XP\_065055150\_4289-model10.1 | -7.88639 |
| # cluster 11 - Size: 1 |  |  |
|  | XP\_065055150\_4289-model11 | -8.22744 |
| # cluster 12 - Size: 15 |  |  |
|  | XP\_065055150\_4289-model12 | -8.07715 |
|  | XP\_065055150\_4289-model12.1 | -7.74612 |
|  | XP\_065055150\_4289-model12.2 | -7.68179 |
|  | XP\_065055150\_4289-model12.3 | -7.68085 |
|  | XP\_065055150\_4289-model12.4 | -7.41848 |
|  | XP\_065055150\_4289-model12.5 | -7.40283 |
|  | XP\_065055150\_4289-model12.6 | -7.35616 |
|  | XP\_065055150\_4289-model12.7 | -7.18228 |
|  | XP\_065055150\_4289-model12.8 | -7.10797 |
|  | XP\_065055150\_4289-model12.9 | -7.10724 |
|  | XP\_065055150\_4289-model12.10 | -7.10115 |
|  | XP\_065055150\_4289-model12.11 | -7.06867 |
|  | XP\_065055150\_4289-model12.12 | -7.00335 |
|  | XP\_065055150\_4289-model12.13 | -6.97417 |
|  | XP\_065055150\_4289-model12.14 | -6.59498 |
| # cluster 13 - Size: 1 |  |  |
|  | XP\_065055150\_4289-model13 | -7.97988 |
| # cluster 14 - Size: 1 |  |  |
|  | XP\_065055150\_4289-model14 | -7.96022 |
| # cluster 15 - Size: 5 |  |  |
|  | XP\_065055150\_4289-model15 | -7.94893 |
|  | XP\_065055150\_4289-model15.1 | -7.73909 |
|  | XP\_065055150\_4289-model15.2 | -7.60979 |
|  | XP\_065055150\_4289-model15.3 | -7.47377 |
|  | XP\_065055150\_4289-model15.4 | -7.42244 |
| # cluster 16 - Size: 6 |  |  |
|  | XP\_065055150\_4289-model16 | -7.87652 |
|  | XP\_065055150\_4289-model16.1 | -7.51427 |
|  | XP\_065055150\_4289-model16.2 | -7.50842 |
|  | XP\_065055150\_4289-model16.3 | -7.13602 |
|  | XP\_065055150\_4289-model16.4 | -7.09637 |
|  | XP\_065055150\_4289-model16.5 | -7.02118 |
| # cluster 17 - Size: 2 |  |  |
|  | XP\_065055150\_4289-model17 | -7.82868 |
|  | XP\_065055150\_4289-model17.1 | -7.21067 |
| # cluster 18 - Size: 6 |  |  |
|  | XP\_065055150\_4289-model18 | -7.69664 |
|  | XP\_065055150\_4289-model18.1 | -7.28083 |
|  | XP\_065055150\_4289-model18.2 | -7.26886 |
|  | XP\_065055150\_4289-model18.3 | -7.24208 |
|  | XP\_065055150\_4289-model18.4 | -7.1539 |
|  | XP\_065055150\_4289-model18.5 | -6.90696 |
| # cluster 19 - Size: 1 |  |  |
|  | XP\_065055150\_4289-model19 | -7.66558 |
| # cluster 20 - Size: 1 |  |  |
|  | XP\_065055150\_4289-model20 | -7.61403 |
| # cluster 21 - Size: 2 |  |  |
|  | XP\_065055150\_4289-model21 | -7.6045 |
|  | XP\_065055150\_4289-model21.1 | -6.61294 |
| # cluster 22 - Size: 3 |  |  |
|  | XP\_065055150\_4289-model22 | -7.60362 |
|  | XP\_065055150\_4289-model22.1 | -6.85122 |
|  | XP\_065055150\_4289-model22.2 | -6.84762 |
| # cluster 23 - Size: 1 |  |  |
|  | XP\_065055150\_4289-model23 | -7.49479 |
| # cluster 24 - Size: 2 |  |  |
|  | XP\_065055150\_4289-model24 | -7.47951 |
|  | XP\_065055150\_4289-model24.1 | -7.41648 |
| # cluster 25 - Size: 1 |  |  |
|  | XP\_065055150\_4289-model25 | -7.47349 |
| # cluster 26 - Size: 1 |  |  |
|  | XP\_065055150\_4289-model26 | -7.44136 |
| # cluster 27 - Size: 1 |  |  |
|  | XP\_065055150\_4289-model27 | -7.41445 |
| # cluster 28 - Size: 1 |  |  |
|  | XP\_065055150\_4289-model28 | -7.39676 |
| # cluster 29 - Size: 6 |  |  |
|  | XP\_065055150\_4289-model29 | -7.36789 |
|  | XP\_065055150\_4289-model29.1 | -7.32479 |
|  | XP\_065055150\_4289-model29.2 | -7.28417 |
|  | XP\_065055150\_4289-model29.3 | -7.16269 |
|  | XP\_065055150\_4289-model29.4 | -6.96053 |
|  | XP\_065055150\_4289-model29.5 | -6.62317 |
| # cluster 30 - Size: 1 |  |  |
|  | XP\_065055150\_4289-model30 | -7.36527 |
| # cluster 31 - Size: 1 |  |  |
|  | XP\_065055150\_4289-model31 | -7.3645 |
| # cluster 32 - Size: 1 |  |  |
|  | XP\_065055150\_4289-model32 | -7.34828 |
| # cluster 33 - Size: 1 |  |  |
|  | XP\_065055150\_4289-model33 | -7.31319 |
| # cluster 34 - Size: 1 |  |  |
|  | XP\_065055150\_4289-model34 | -7.28807 |
| # cluster 35 - Size: 1 |  |  |
|  | XP\_065055150\_4289-model35 | -7.20723 |
| # cluster 36 - Size: 1 |  |  |
|  | XP\_065055150\_4289-model36 | -7.16178 |
| # cluster 37 - Size: 1 |  |  |
|  | XP\_065055150\_4289-model37 | -7.15643 |
| # cluster 38 - Size: 2 |  |  |
|  | XP\_065055150\_4289-model38 | -7.1532 |
|  | XP\_065055150\_4289-model38.1 | -7.12854 |
| # cluster 39 - Size: 1 |  |  |
|  | XP\_065055150\_4289-model39 | -7.14664 |
| # cluster 40 - Size: 1 |  |  |
|  | XP\_065055150\_4289-model40 | -7.12225 |
| # cluster 41 - Size: 2 |  |  |
|  | XP\_065055150\_4289-model41 | -7.10743 |
|  | XP\_065055150\_4289-model41.1 | -6.86664 |
| # cluster 42 - Size: 1 |  |  |
|  | XP\_065055150\_4289-model42 | -7.10735 |
| # cluster 43 - Size: 1 |  |  |
|  | XP\_065055150\_4289-model43 | -7.10486 |
| # cluster 44 - Size: 1 |  |  |
|  | XP\_065055150\_4289-model44 | -7.10149 |
| # cluster 45 - Size: 1 |  |  |
|  | XP\_065055150\_4289-model45 | -7.03852 |
| # cluster 46 - Size: 1 |  |  |
|  | XP\_065055150\_4289-model46 | -7.02252 |
| # cluster 47 - Size: 1 |  |  |
|  | XP\_065055150\_4289-model47 | -6.98441 |
| # cluster 48 - Size: 1 |  |  |
|  | XP\_065055150\_4289-model48 | -6.97293 |
| # cluster 49 - Size: 1 |  |  |
|  | XP\_065055150\_4289-model49 | -6.9284 |
| # cluster 50 - Size: 1 |  |  |
|  | XP\_065055150\_4289-model50 | -6.9223 |
| # cluster 51 - Size: 1 |  |  |
|  | XP\_065055150\_4289-model51 | -6.91321 |
| # cluster 52 - Size: 1 |  |  |
|  | XP\_065055150\_4289-model52 | -6.87849 |
| # cluster 53 - Size: 1 |  |  |
|  | XP\_065055150\_4289-model53 | -6.78813 |
| # cluster 54 - Size: 1 |  |  |
|  | XP\_065055150\_4289-model54 | -6.76541 |
| # cluster 55 - Size: 1 |  |  |
|  | XP\_065055150\_4289-model55 | -6.75529 |
| # cluster 56 - Size: 1 |  |  |
|  | XP\_065055150\_4289-model56 | -6.6234 |
|  |  |  |
